# Supplementary material for: Meta-Analysis and Gene Set Enrichment Relative to ER Status Reveal Elevated Activity of MYC and E2F in the “Basal” Breast Cancer Subgroup
Source: PLoS One. 2009 Mar 9;4(3):e4710. doi: 10.1371/journal.pone.0004710 (PMC2650420; doi:10.1371/journal.pone.0004710)
Supplement: Dataset S1 — GSEA results from first screen. The file contains a zipped archive of the HTML results of the second GSEA screen. Reports for enriched or depleted datasets may be accessed from the “index.html” link within the my_analysis.GseaPreranked.1219975950625.rpt.zip ZIP archive. Enrichment plots are available for each of the 50 most enriched and depleted gene sets. (13.32 MB ZIP) [file pone.0004710.s001.zip › my_analysis.GseaPreranked.1219975950625.rpt.zip!//CGGAARNGGCNG_UNKNOWN.html]

Details for gene set CGGAARNGGCNG\_UNKNOWN[GSEA]

|  || Dataset | zSco.ERneg.to.ERpos\_collapsed |
| Phenotype | NoPhenotypeAvailable |
| Upregulated in class | na\_neg |
| GeneSet | CGGAARNGGCNG\_UNKNOWN |
| Enrichment Score (ES) | -0.45321062 |
| Normalized Enrichment Score (NES) | -1.6782849 |
| Nominal p-value | 0.0080 |
| FDR q-value | 0.07436283 |
| FWER p-Value | 0.986 |
Table: GSEA Results Summary

  

Fig 1: Enrichment plot: CGGAARNGGCNG\_UNKNOWN      
 Profile of the Running ES Score & Positions of GeneSet Members on the Rank Ordered List

  

| PROBE | GENE SYMBOL | GENE\_TITLE | RANK IN GENE LIST | RANK METRIC SCORE | RUNNING ES | CORE ENRICHMENT || 1 | IRAK1 |  |  | 553 | 4.230 | 0.0299 | No |
| 2 | UMPS |  |  | 3124 | 1.877 | -0.1318 | No |
| 3 | ATP2A2 |  |  | 3337 | 1.777 | -0.1178 | No |
| 4 | WWP2 |  |  | 3365 | 1.765 | -0.0900 | No |
| 5 | SLC36A1 |  |  | 3997 | 1.512 | -0.1119 | No |
| 6 | PANK2 |  |  | 4168 | 1.445 | -0.1003 | No |
| 7 | EML2 |  |  | 5249 | 1.064 | -0.1636 | No |
| 8 | TMEM30A |  |  | 5326 | 1.036 | -0.1518 | No |
| 9 | GANAB |  |  | 6230 | 0.738 | -0.2073 | No |
| 10 | GTF2A1 |  |  | 6460 | 0.664 | -0.2133 | No |
| 11 | POLH |  |  | 6522 | 0.642 | -0.2071 | No |
| 12 | C9ORF74 |  |  | 6593 | 0.624 | -0.2018 | No |
| 13 | CDC37 |  |  | 7560 | 0.284 | -0.2697 | No |
| 14 | CCDC6 |  |  | 7584 | 0.276 | -0.2668 | No |
| 15 | TP53 |  |  | 7848 | 0.167 | -0.2837 | No |
| 16 | LMAN2 |  |  | 8003 | 0.104 | -0.2936 | No |
| 17 | CLTC |  |  | 8682 | -0.162 | -0.3419 | No |
| 18 | BIN3 |  |  | 8736 | -0.184 | -0.3427 | No |
| 19 | TPT1 |  |  | 8852 | -0.232 | -0.3475 | No |
| 20 | PAX2 |  |  | 9439 | -0.534 | -0.3826 | No |
| 21 | SPTLC2 |  |  | 9646 | -0.642 | -0.3872 | No |
| 22 | RAB4B |  |  | 10524 | -1.188 | -0.4331 | Yes |
| 23 | DUSP6 |  |  | 10680 | -1.305 | -0.4227 | Yes |
| 24 | HNRPH1 |  |  | 10825 | -1.410 | -0.4098 | Yes |
| 25 | CFL1 |  |  | 10849 | -1.430 | -0.3873 | Yes |
| 26 | N-PAC |  |  | 11552 | -2.088 | -0.4049 | Yes |
| 27 | EGR1 |  |  | 11628 | -2.148 | -0.3742 | Yes |
| 28 | HTATIP |  |  | 11650 | -2.166 | -0.3392 | Yes |
| 29 | RHOBTB2 |  |  | 11904 | -2.440 | -0.3170 | Yes |
| 30 | CXORF12 |  |  | 12144 | -2.762 | -0.2883 | Yes |
| 31 | VPS39 |  |  | 12326 | -2.994 | -0.2513 | Yes |
| 32 | RNF14 |  |  | 12711 | -3.640 | -0.2187 | Yes |
| 33 | TAF9 |  |  | 12778 | -3.813 | -0.1592 | Yes |
| 34 | RAD17 |  |  | 13147 | -5.016 | -0.1022 | Yes |
| 35 | RGL2 |  |  | 13304 | -6.810 | 0.0012 | Yes |
Table: GSEA details [plain text format]

  

Fig 2: CGGAARNGGCNG\_UNKNOWN: Random ES distribution      
 Gene set null distribution of ES for **CGGAARNGGCNG\_UNKNOWN**
